# Sarcomatoid Variant of Urothelial Carcinoma of the Renal Pelvis with Inferior Vena Cava Tumour Thrombus: A Case Report and Literature Review

**DOI:** 10.1155/2018/1837510

**Published:** 2018-01-17

**Authors:** Sameera Rashid, Mohammed Akhtar

**Affiliations:** Department of Laboratory Medicine and Pathology, Hamad Medical Corporation, Doha, Qatar

## Abstract

Sarcomatoid variant of urothelial carcinoma (SVUC) of the renal pelvis is a rare entity. To the best of our knowledge, around 25 cases of this neoplasm have been reported in the literature to date, most of which were of high stage. The inferior vena cava tumour thrombus, which is a hallmark of renal cell carcinoma (RCC), may rarely be found in urothelial carcinoma of renal pelvis. In this report, a case of SVUC associated with tumour extension to inferior vena cava is documented. This association has been encountered in only one previously reported case. The possibility of urothelial carcinoma of the renal pelvis should therefore be included in the differential diagnosis of tumour thrombus of the inferior vena cava.

## 1. Introduction

Carcinoma of the kidney and renal pelvis represents the eighth most common cancer in the United States with an estimated 63,990 new cases and 14,400 deaths by renal tumours in the year 2017 [1]. Urothelial carcinoma (UC) accounts for 10 to 15% of all primary renal malignancies, with the most common malignant tumour being renal cell carcinoma (RCC). Inferior vena cava tumour thrombus, which is a hallmark of RCC, is rarely found in urothelial carcinoma. Similar vena cava tumour thrombi, however, are found in less common retroperitoneal primary neoplasms such as Wilms' tumour and various adrenal, uterine, and urinary bladder tumours [2].

Sarcomatoid variant of urothelial carcinoma (SVUC) of the renal pelvis is a rare tumour with aggressive clinical behaviour [3]. Around twenty-five cases of SVUC arising from the renal pelvis have been reported [4–12]. Of these only one presented with tumour thrombus in the inferior vena cava. We report one more case of SVUC with involvement of the inferior vena cava.

## 2. Case Report

A 70-year-old male presented with urgency, urinary incontinence, and nocturia for five days. The patient was known to have benign prostatic hyperplasia (on Tamsulosin) and had a long- standing history of anaemia. No palpable abdominal mass was detected during the physical examination. His laboratory evaluation revealed anaemia (haemoglobin: 8.5 mg/dl, MCV: 80 fl, MCH 24.8 pg, MCHC 30.9 gm/dl, WBC 14 × 10^3^/microliter, creatinine 90 micromole/dl, and platelet 156 ng/ml).

**Figure 1:**
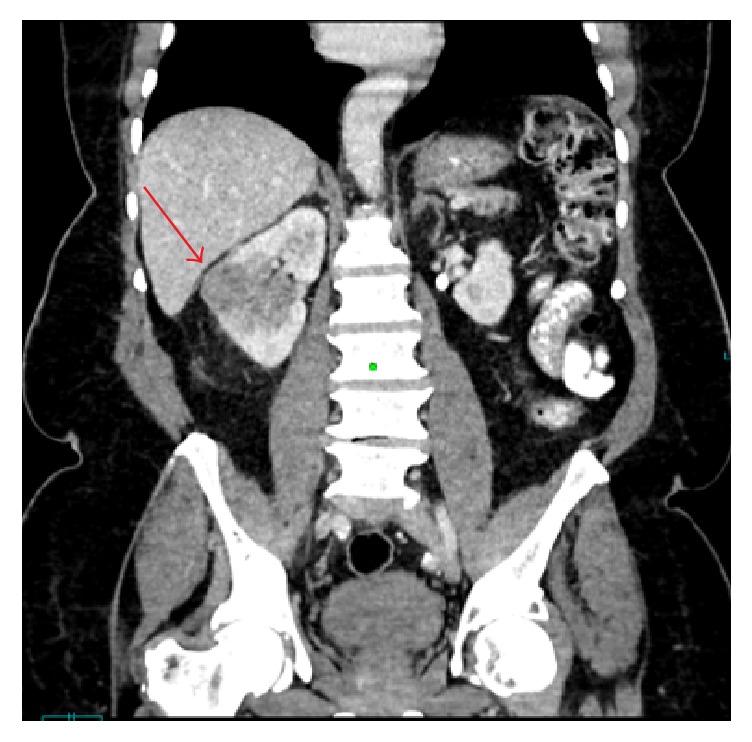
Computed tomography with contrast (CT) coronal image demonstrates a heterogeneously enhancing hypodense right renal mid pole mass (red arrow) occupying the renal cortex and renal pelvis with mild surrounding fat stranding.

**Figure 2:**
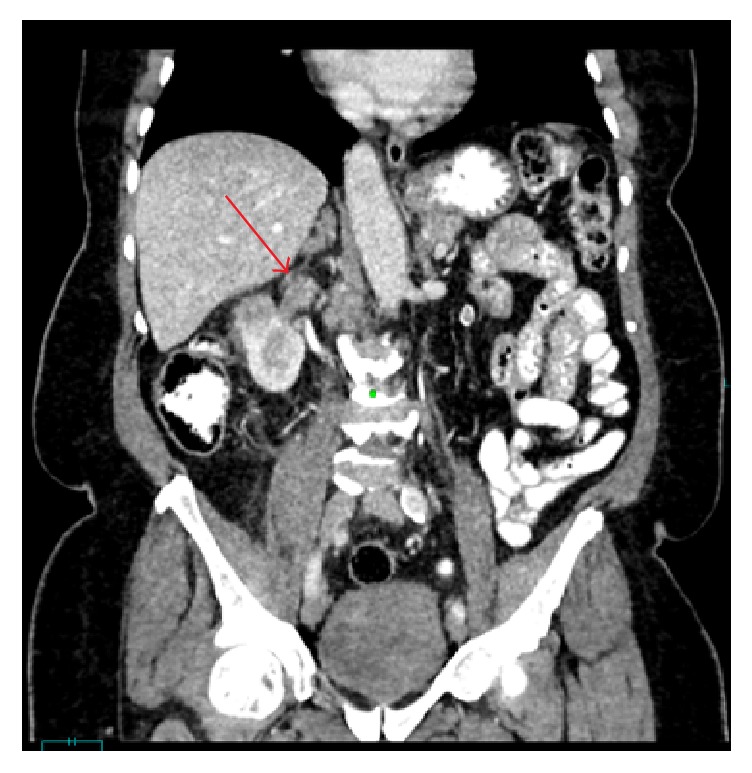
Contrast enhanced computed tomography coronal image demonstrates tumoural thrombus within the right renal vein (red arrow).

Ultrasound of the renal system showed a right renal mass. An abdominal computed tomography (CT) revealed an ill-defined infiltrative 4 cm renal mass in the mid to lower pole region of the right kidney, involving the renal parenchyma, renal hilum, and the renal pelvis (Figure 1). Similar findings were also noted on a magnetic resonance imaging (MRI) which also revealed a tumour thrombus extending along the entire course of the right renal vein and projecting into the right side of the lumen of the inferior vena cava (Figure 2).

A right laparoscopic radical nephroureterectomy with thrombectomy of the inferior vena cava was performed. The right kidney was enlarged, solid, and adherent to perirenal adipose tissue. The middle and lower part of right ureter were dilated. The thrombus was gently milked from the inferior vena cava and removed separately.

**Figure 3:**
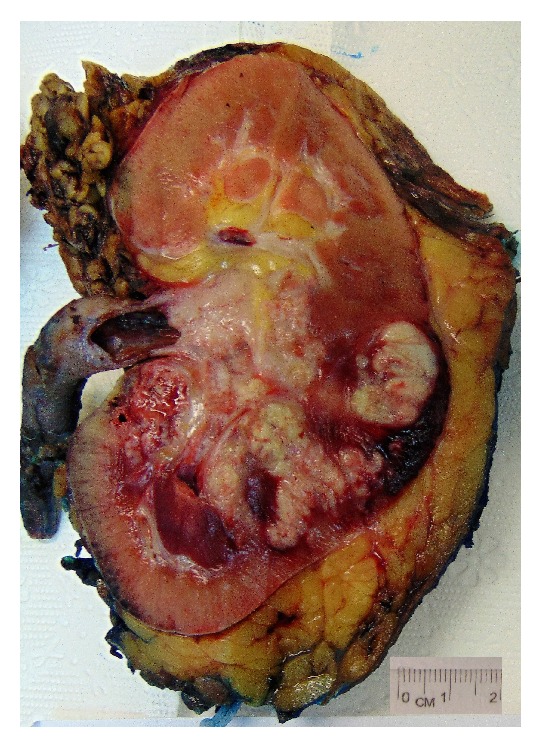
Right kidney with attached ureter showing tumour arising from the renal pelvis projecting into the proximal ureter and infiltrating the renal parenchyma.

Gross examination of the nephrectomy specimen showed a 5.5 × 4.5 × 4.5 cm greyish tan friable mass arising in the renal pelvis, projecting into the proximal ureter, and extensively infiltrating the adjacent renal parenchyma (Figure 3).

**Figure 4:**
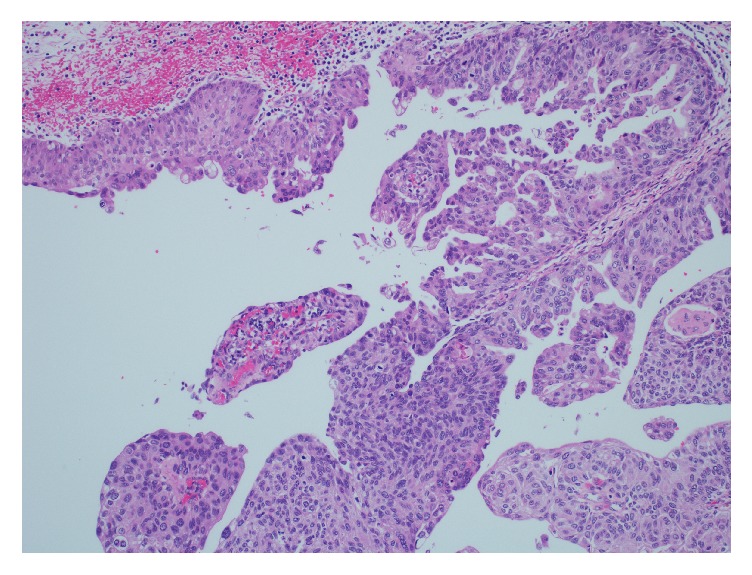
A low power view of the papillary urothelial carcinoma projecting into the renal pelvis. Carcinoma in situ is present in the adjacent urothelial mucosa.

Histological examination revealed a high grade urothelial carcinoma with sarcomatoid differentiation. The tumour was arising in the renal pelvis with infiltration of the renal parenchyma and extension into renal sinus and the renal vein. Carcinoma in situ involving the adjacent urothelium was noted (Figure 4).

**Figure 5:**
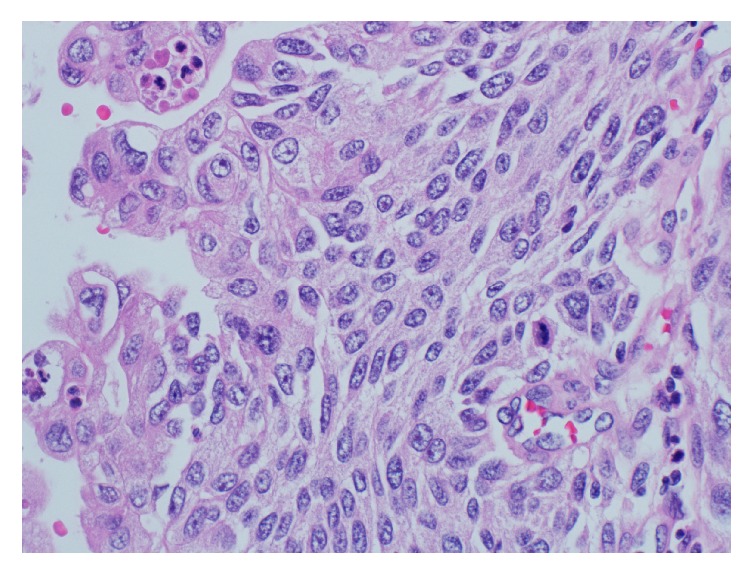
Photomicrograph depicting high grade urothelial carcinoma with transformation to sarcomatoid carcinoma characterized by a spindle cell pattern.

**Figure 6:**
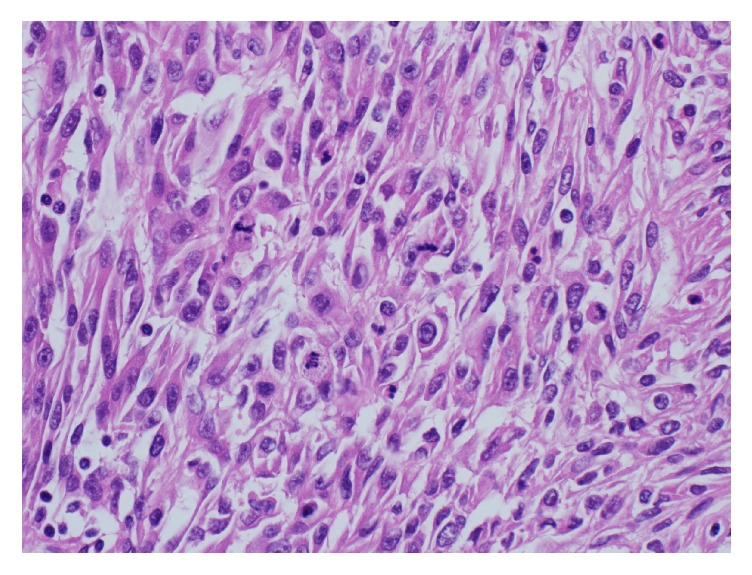
Higher magnification photomicrograph featuring sarcomatoid component with spindle proliferation, nuclear pleomorphism, and increased mitotic activity.

The sarcomatoid elements were composed of spindle shaped cells with marked nuclear pleomorphism and high mitotic activity (Figures 5 and 6).

**Figure 7:**
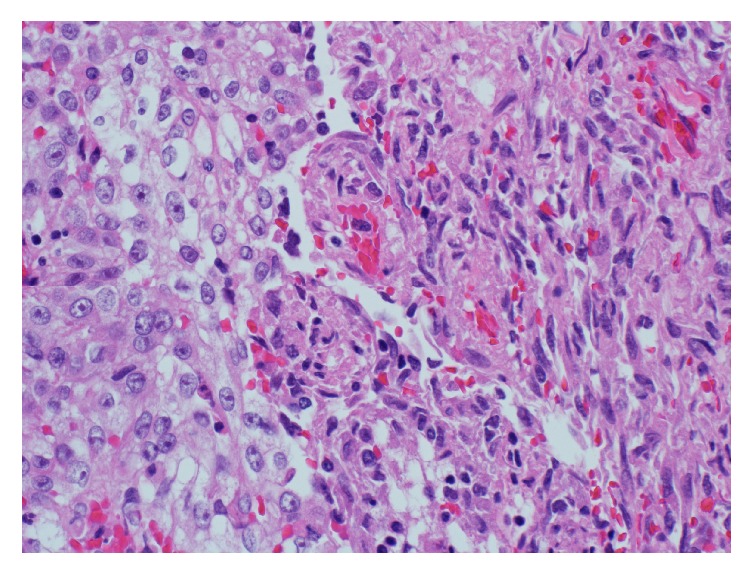
Tumour thrombus from the inferior vena cava with high grade urothelial carcinoma and the sarcomatoid component.

Extensive areas of tumour necrosis were also seen. The resection margin of the ureter was free of tumour. The tumour thrombus from the inferior vena cava was composed of high grade urothelial carcinoma along with the sarcomatoid component (Figure 7).

On immunohistochemistry the carcinoma component was positive for CK7, CK20, p63, p53, and GATA-3, while the sarcomatous component stained strongly and diffusely positive for vimentin. Both components did not show any staining for desmin or smooth muscle actin (Table 1).

## 3. Discussion

Renal cell carcinoma (RCC) is known to have a predisposition for vascular invasion, and in 10–25% cases tumour thrombus may extend into the inferior vena cava [13]. However, urothelial carcinoma arising from the renal pelvis rarely invades IVC. Although 25 cases of IVC involvement by urothelial carcinoma are reported in the English literature, there has been only one previously reported case of SVUC extension into IVC [14].

Sarcomatoid variant of urothelial carcinoma is an aggressive but uncommon type of urothelial carcinoma with around 100 reported cases, an overwhelming majority of which arise in the urinary bladder [15–17]. In the literature, these tumours have also been designated as carcinosarcoma and spindle cell carcinoma [17]. Lopez-Beltran et al. [6] stated that microscopically sarcomatoid urothelial carcinoma contains a urothelial, glandular, or small cell component with variable degrees of differentiation and that carcinoma in situ is found in 30% of these cases. Occasionally carcinoma in situ is the only identifiable epithelial component in these cases. Heterologous mesenchymal sarcomatoid elements may occasionally be encountered in SVUC, with most common component being osteosarcoma followed by chondrosarcoma components.

Two opposing theories have been proposed regarding the pathogenesis of SVUC. The monoclonal theory states that the carcinomatous and sarcomatous tumour cells are both derived from a single pluripotent stem cell. The multiclonal theory, on the other hand, states that the sarcomatoid carcinoma is a collision tumour composed of the derivatives of two or more stem cells of separate epithelial and mesenchymal origin [18, 19]. Eble et al. stated that sarcomatoid variant of urothelial carcinoma is the most appropriate terminology that should be used for all biphasic malignant neoplasms which exhibit morphologic and/or immunohistochemical evidence of epithelial and mesenchymal differentiation with or without heterologous elements [4]. The WHO classification system acknowledges the controversy surrounding this issue but refers to these lesions as sarcomatoid variant of urothelial carcinoma [4].

Sarcomatoid variant of urothelial carcinoma of the renal pelvis is a rare entity with approximately 25 reported cases. A poor prognosis has been reported for most patients, and only a few patients have survived for more than two years after diagnosis [4–12]. To the best of our knowledge, inferior vena cava tumour thrombus in a patient with SVUC of renal pelvis has been documented only once before in a case reported by Wick and Swanson [20]. This was a 61-year-old man who presented with a right renal mass with a vena caval thrombus, demonstrated on computed tomography, that was radiologically diagnosed as renal cell carcinoma. The results of routine laboratory examinations and urinalysis were within normal limits. Preoperative planning was critical owing to the presence of the vena caval thrombus. A radical nephrectomy, vena caval thrombectomy, and regional lymphadenectomy were done. The pathologic report was consistent with a high grade, invasive urothelial carcinoma, with sarcomatoid differentiation arising in the renal pelvis and involving the renal vein and inferior vena cava. Our report is the second case of SVUC of the renal pelvis with inferior vena cava tumour thrombus. Since vena caval involvement is usually associated with renal cell carcinoma, this finding in the CT scan may lead to an erroneous impression of renal cell carcinoma.

The differential diagnosis of SVUC includes primary renal sarcoma and renal cell carcinoma with sarcomatoid differentiation. Both lesions are rare. Adult renal sarcoma accounts for 0.8% of renal cancer cases and generally has a poor prognosis [21]. RCC with sarcomatoid features is also associated with a poor prognosis, but unlike renal carcinoma targeted therapy has no clear benefits [22]. Treatment of inferior vena cava tumour thrombus should be based on the primary pathology and natural behaviour of a given tumour in a specific patient rather than only focusing on the level and extent of venous invasion [21].

## 4. Conclusion

A rare case of SVUC arising in the renal pelvis with inferior vena cava tumour thrombus is documented. As inferior vena cava tumour thrombus is more common with renal cell carcinoma, these cases are usually diagnosed preoperatively as renal cell carcinoma. However, as this case demonstrates, renal vein and inferior vena cava involvement may also be seen in urothelial carcinomas arising in the renal pelvis.

## Figures and Tables

**Table 1:** Comparison of immunohistochemistry findings in the sarcomatous and urothelial components.

Antibody	Carcinoma component	Sarcomatoid component
CK 7	Diffusely positive, strong intensity	Weak, focal positive
CK 20	Focally positive, strong intensity	Negative
p53	Focally positive, strong intensity	Negative
P63	Diffusely positive, strong intensity	Negative
GATA-3	Diffusely positive, strong intensity	Weak, focal positive
Vimentin	Negative	Diffusely positive, strong intensity
Desmin	Negative	Negative
Smooth muscle action	Negative	Negative
